# Recent Advances in Conjugation Chemistry Expanding the Applications of the DNA Tetrahedron

**DOI:** 10.1111/cbdd.70369

**Published:** 2026-08-04

**Authors:** Tyler J. Rutherford, Christopher J. Wilds

**Affiliations:** ^1^ Department of Chemistry and Biochemistry Concordia University Montréal Québec Canada

## Abstract

The tetrahedral DNA nanostructure (TDN) has emerged as a premier, highly programmable framework for targeted drug delivery and diagnostics. This review provides a definitive, chemistry‐first design guide for TDN engineering, systematically structured across three translational tiers. First, we examine foundational derivatives, detailing how precise modifications across the three components of the nucleotide scaffold confer essential nuclease resistance and structural longevity. Second, a diverse array of conjugation strategies is compiled, mapping the chemical mechanics of stable and responsive covalent linkages alongside distinct non‐covalent loading modalities, including intercalation, metallo‐coordination, and groove binding. Third, we explore advanced derivatives for materials science, detailing how merging TDNs with traditional nanotechnology or smart polymeric matrices yields hybrid architectures engineered into sophisticated analytical tools and biomaterials. Advancing this platform will entail integration of AI‐driven computational models and navigating critical physiological barriers, establishing the functionalized TDN as a definitive cornerstone of next‐generation nanomedicine.

## Graphical abstract

The tetrahedral DNA nanostructure (TDN) is a promising drug delivery and biosensing platform that can be chemically modified with absolute payload precision. This review highlights explicit conjugation chemistries driving functionalization of the TDN for a broad range of applications, advancing its role in support of next‐generation biomimetic nanomedicine.

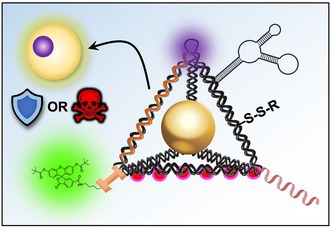

## 1 Introduction

**FIGURE 1:**
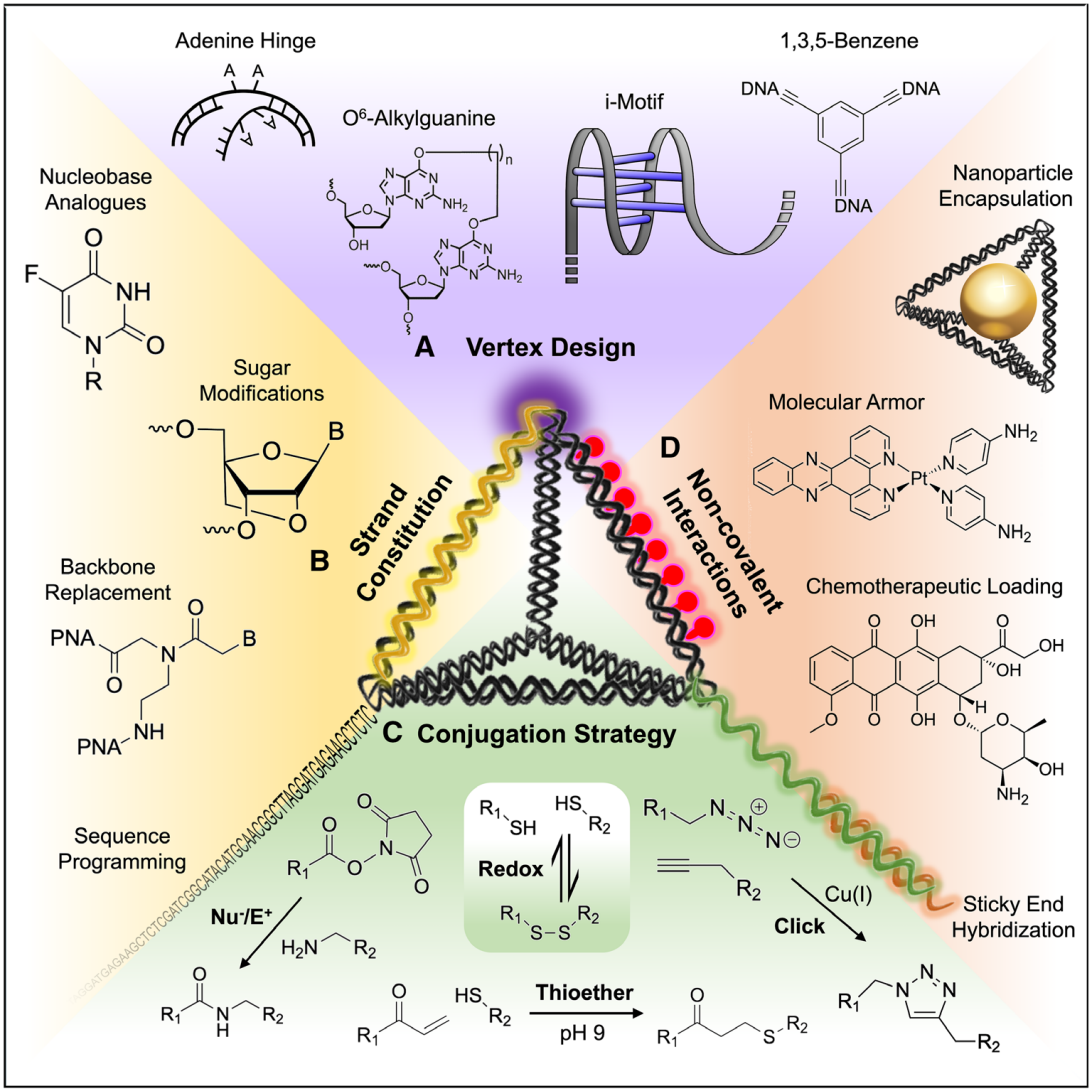
Design space of the synthetic DNA tetrahedron. (A) Vertex design—defines shape, rigidity, and potential reactivity of the structure. (B) Strand constitution—nucleobase, sugar, backbone, and sequence composition can engineer helix structure, charge, and nuclease stability. (C) Conjugation strategy—enables post‐synthetic functionalization employing stable or dynamic covalent linkages. (D) Non‐covalent interactions—host–guest interactions that encapsulate or intercalate cargo.

Nucleic acids are renowned for their ability to store information in the canonical Watson–Crick base pairing between complementary purines and pyrimidines. Beyond encoding genetic information for protein synthesis, DNA provides unique structural programmability. The advent of solid‐phase oligonucleotide synthesis allowed scientists to generate these biopolymers with defined sequences and high purity (Roy and Caruthers 2013; Alvarado‐Urbina et al. 1981). The pioneering work of Nadrian Seeman and his first account of topologically complex 2D and 3D nano‐frameworks occurred in the 1980s (Seeman and Sleiman 2017; Chen and Seeman 1991; Seeman and Belcher 2002). Amongst the elegant architectures that have evolved from these landmark studies, the tetrahedral DNA nanostructure (TDN) stood out for its facile one‐pot self‐assembly (Goodman et al. 2004), structural rigidity (Goodman et al. 2005), and cellular uptake capabilities (Walsh et al. 2011; Liang et al. 2014; Xia et al. 2016). While a diverse array of TDNs have since been developed to tackle challenges found in targeted drug delivery and bioanalytical applications (Gu et al. 2025), their fundamental efficacy hinges on the method of payload attachment. This review diverges from the traditional applications‐driven narrative, focusing on the explicit conjugation chemistries that enabled these advancements. Going beyond standard couplings, we systematically discuss novel approaches including dynamic disulfide bridges (Chen et al. 2024; Pontarelli et al. 2022), non‐native nucleic acids (Copp and Wilds 2020; Jorge et al. 2018), intercalation (Joaqui‐Joaqui et al. 2025; Kim et al. 2013), and click chemistry (Xia et al. 2016; Wang et al. 2020; Valsangkar et al. 2019) to provide a definitive design guide for precise cargo loading (Figure 1). The current literature has moved beyond initial proof‐of‐concept designs, overcoming complex barriers toward clinical translation. Critical hurdles including scalable synthesis (Praetorius et al. 2017; Halley et al. 2019), tissue targeting (Wang et al. 2019; Zimmermann et al. 2017; Debacker et al. 2020), cytosolic delivery (Tang et al. 2025; Liu et al. 2024; Yang et al. 2024; Zhou et al. 2025; Kim et al. 2025), and endosomal escape (Liang et al. 2014; Han et al. 2021; He et al. 2024; Muro 2014) are no longer mere biological challenges, but engineering problems requiring sophisticated nanoarchitectural design. The TDN merges a robust payload capacity akin to lipid nanoparticles with the biomimetic precision of a viral capsid and chemical modifiability of a synthetic nanoparticle. Ultimately, the TDN unifies the disparate paradigms, as an exceptionally versatile hybrid chassis, uniquely equipped to redefine the landscape of targeted therapeutics.

### 1.1 Historic Precedent and Synthesis

To appreciate the current advancements of TDNs, we must contextualize the evolution of this nanotechnology. The pivotal breakthrough occurred in 2004 when Goodman et al. established the one‐pot thermal annealing protocol for the TDN. Prior methods necessitated arduous ligation steps to construct artificial DNA frameworks in low yield (Chen and Seeman 1991; Zhang and Seeman 1994). Their innovation lay in the thermodynamic favorability of TDN assembly through precise sequence design. By combining then heating four equimolar complementary oligonucleotides, followed by controlled cooling, the desired TDN assembled in solution with > 90% yield (Goodman et al. 2004). Recognizing the scalability of this method, the field quickly pivoted to complex functionalization for analytical and biomedical applications.

**FIGURE 2:**
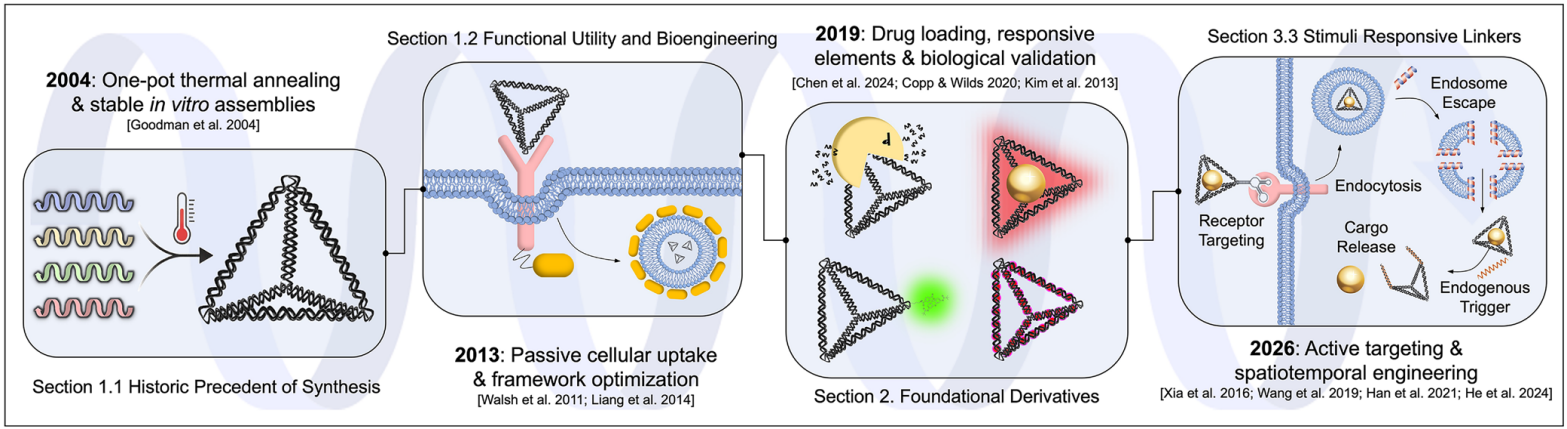
Timeline depicting the evolution of TDN carriers from structural marvels to advanced biological delivery systems. The TDN has leveraged novel conjugation chemistries and fundamental biology to bridge nanotechnology into a responsive nanoparticle, drug carrier, and injectable diagnostic.

This original study established decades of exploration, defined by rigorous biological validation and functional diversification (Figure 2). Seminal papers during this period demonstrated that TDNs could spontaneously enter mammalian cells without the need for transfection agents (Walsh et al. 2011; Liang et al. 2014; Xia et al. 2016). A 2019 study by Lacroix et al. challenged this notion, highlighting that degradation artifacts often confound uptake signals, suggesting the adoption of more rigorous controls in future uptake studies (Raniolo et al. 2019). Methods such as cell‐surface anchors (Wang, Chopra, et al. 2024), hydrophobic handles (Singh et al. 2023), and enzymatic shielding (Joaqui‐Joaqui et al. 2025; Gerling et al. 2018) have become essential pillars of efficacy in novel TDN design. Conjugation methods have developed in parallel, with researchers consistently discovering innovative approaches. The development phase laid the groundwork for complex systems that shift the focus from passive structural uptake to active targeting (Figure 2).

### 1.2 Functional Utility and Bioengineering

The TDN is increasingly recognized for its potential as a modular bioengineering platform in drug delivery and biosensing. Beyond its utility as a carrier, the TDN itself exhibits intrinsic anti‐inflammatory and antioxidant properties, influencing cellular behaviors and immune responses (Zhang, Lin, et al. 2018). To expand its therapeutic scope, novel conjugation strategies leveraging modified edges (Ge et al. 2024; Zhang et al. 2022; Zhang, Ma, et al. 2018) and vertices (Han et al. 2021; Zhang et al. 2020; Wu, Zhu, et al. 2024) have emerged. Each nucleotide serves as a precise anchor point for a diverse library of functional moieties. For example, conjugating aptamers to the vertex effectively transforms the basal scaffold into a receptor‐mediated homing device (Shiu et al. 2018). Furthermore, the rigid biocompatible cage provides a reservoir for encapsulating metallic nanoparticles (Wu, Fu, et al. 2024; Ma and Miao 2019; Tang et al. 2021) or intercalating small molecules (Xia et al. 2016; Kim et al. 2013; Kang et al. 2017). The TDN sequence can also function as a therapeutic with antisense oligonucleotides (ASOs) or siRNA directly integrated to target intracellular machinery (Chen et al. 2024; Zhou et al. 2021; Li et al. 2023). By integrating conjugates responsive to endogenous and exogenous stimuli, these systems can be engineered to target‐specific tissues or organelles (Chandrasekaran and Halvorsen 2019). Explicit triggers such as pH (Liu et al. 2013; Keum and Bermudez 2012), light (Valsangkar et al. 2019; Han et al. 2011), chemical environment (Chandrasekaran and Halvorsen 2019; Dohno et al. 2011), and enzymatic activity (Copp and Wilds 2020; Ko et al. 2020) precisely interact with the TDN, inducing molecular‐level responses. Consequently, this review will examine recent advancements in TDN conjugation strategies, serving as a framework for future innovations in nanomedicine and bioengineering.

## 2 Foundational Derivatives

### 2.1 Solid‐Phase Synthesis

**FIGURE 3:**
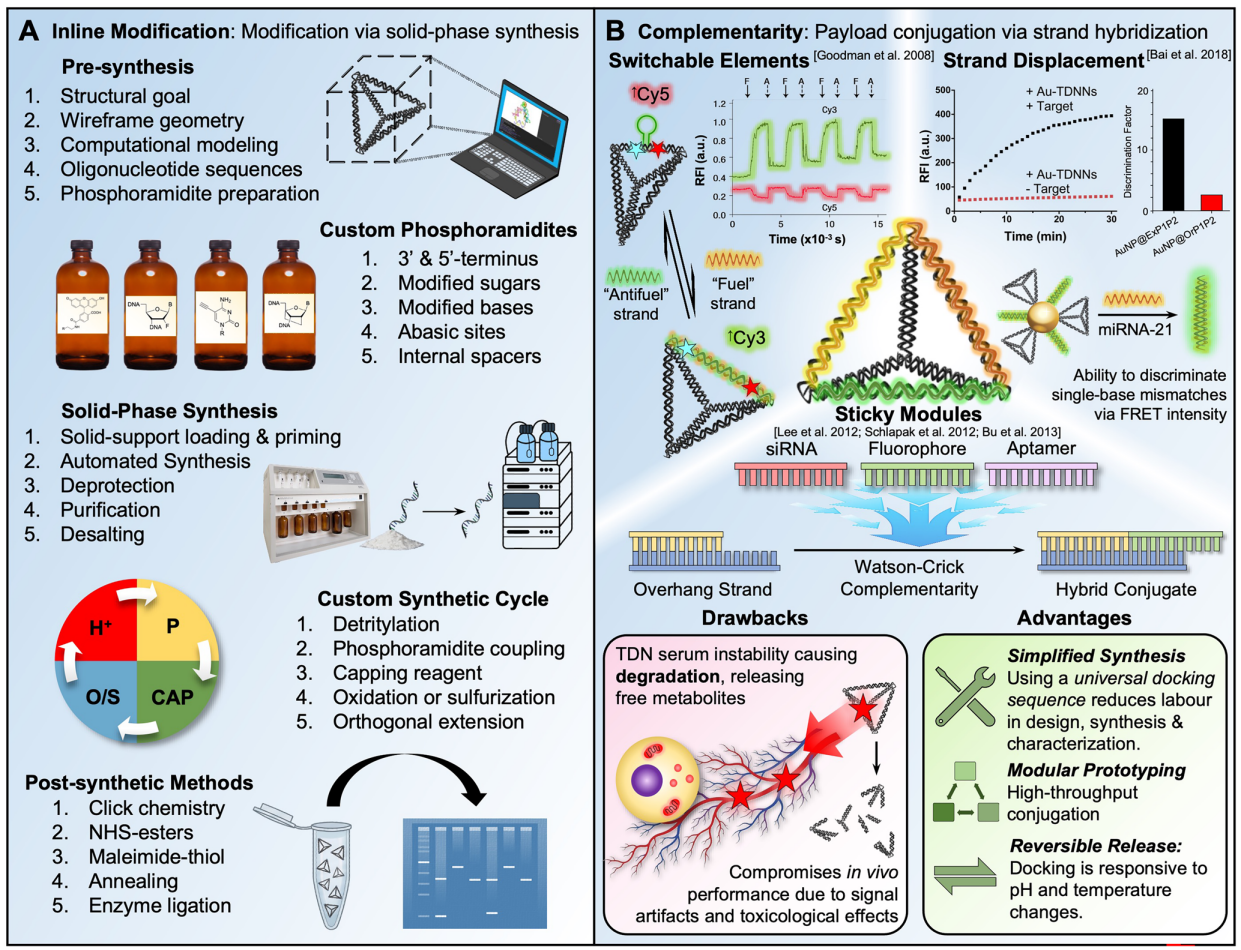
Conceptual pipeline for the design, synthesis, and mechanistic evaluation of sequence‐responsive tetrahedral DNA nanostructures. (A) Workflow for the preparation of responsive nanocarriers capable of triggered cargo release. Strategic sequence design begins with the selection of custom phosphoramidites which directly program the post‐synthetic attributes of the assembled TDN. Integrated modifications must exhibit robust chemical stability to withstand automated synthesis and purification conditions. (B) Single‐stranded oligonucleotides present modular docking sites for complementary payload strands. The non‐covalent hydrogen bonds are susceptible to dissociation by pH, temperature, competing strands, and serum nucleases.

Solid‐phase synthesis remains the foundational strategy for functionalizing TDNs; enabling the seamless, automated incorporation of 5′‐terminal fluorophores (Didenko 2001; Zhong et al. 2018; Theisen et al. 1992), C6 thiol‐modifiers (Gaur 1991; Kupihár et al. 2003), and polyethylene glycol (PEG) (Lu and Zhang 2018; Winkler 2015) via phosphoramidite chemistry. This direct approach is widely adopted by nucleic acid chemists as a robust and readily available technique (Figure 3A) for labeling and derivatizing oligonucleotides (Bergamini et al. 2014; Lietard et al. 2019). However, the utility of this in‐line method is fundamentally compromised by the susceptibility of the unprotected backbone to cleavage by extracellular nucleases and acidic environments (Lacroix et al. 2019). Degradation products generate misleading in vitro cellular uptake profiles that falsely imply the intact TDN is reaching the cytosol (Walsh et al. 2011; Lacroix et al. 2019). TDN complexes release free dye upon degradation, frequently creating fluorescent artifacts in lysosomes and later mitochondria that mimic intact TDNs. Consequently, while solid‐phase synthesis remains the most direct modification route, data derived from these constructs must be strictly validated against degradation controls to differentiate true cytosolic TDN delivery from free dye uptake (Lacroix et al. 2019).

### 2.2 Hybridization and Sticky Ends

The defining feature of DNA nanotechnology is sequence complementarity, utilizing Watson–Crick hybridization to generate complex, rigid architectures. Beyond the ability to construct novel 2D and 3D frameworks, this programmability allows for the integration of intrinsic docking sites via extension of component oligonucleotides into single‐stranded overhangs. This “plug‐and‐play” strategy enables the facile attachment of small interfering RNA (siRNA) (Lee et al. 2012), fluorescent probes (Schlapak et al. 2012), or targeting aptamers (Bu et al. 2013) without the need for exogenous chemical cross‐linkers (Figure 3B). However, oligonucleotide hybridization introduces a significant vulnerability: exposed single‐stranded overhangs are primary substrates for rapid hydrolysis by serum exonucleases (Desai and Shankar 2003). Furthermore, they are sensitive to localized thermodynamic and pH fluctuations in the bloodstream, inducing premature dissociation if the payload is not sufficiently stabilized (Conway et al. 2013; Longmire et al. 2008). To diminish the volatility, recent designs have incorporated toehold clamps (Nguyen et al. 2019; Faheem et al. 2022), extended GC‐rich sequences (Huang et al. 2024), or hairpin shielding (Goodman et al. 2008; Li et al. 2019) to protect the docking site until intracellular biomarkers trigger strand displacement.

Crucially, this vulnerability can be reversed into an elegant mechanism for controlled cargo release. Zhu et al. created a trifunctional TDN‐based nanoprobe with fluorescently labeled hairpins at the vertices. Utilizing the principles of structural complementarity, they formed pH‐responsive Hoogsteen‐bonded triplexes that dissociate upon reaching the acidic tumor microenvironment. Following activation, *survivin* mRNA expressed within cancer cells initiates a cascade of complementary hybridization interactions within the liberated hairpins, yielding a detectable FRET signal (Zhu et al. 2019).

To resolve the challenges of premature payload release, recent protocol extensions have established dynamic, stimuli‐responsive delivery systems utilizing tetrahedral DNA skeletons. By introducing an RNase H‐sensitive DNA–RNA hybrid sequence as a localized bioswitch, these updated frameworks can undergo controlled intracellular unloading of microRNA (miRNA) mimics or inhibitors. These configurations either append the RNA cargo externally to reduce synthesis costs or embed the regulators directly within the tetrahedral topology to shield the payload and maximize tissue penetration (Li et al. 2025).

Shifting the focus from external overhangs to *internal framework complementarity*, modular DNA nanotubes of controlled length were developed utilizing repeating triangular rungs assembled on a linear template (Lo et al. 2010; Rahbani et al. 2015; Platnich et al. 2018). By sealing this framework with complementary linking strands, 20 nm gold nanoparticles can be physically encapsulated within the internal cavities of the resulting nanotubes (Lo et al. 2010). The closed state of the framework relies entirely on the structural integrity of one specific internal linking strand at the cage junctions. On exposure to specific endogenous strands or cellular oligonucleotide biomarkers that possess exact complementary sequences to these internal nodes, a competitive strand displacement reaction is initiated. The resulting duplex dissociates from the complex, systematically “unzipping” the framework back into its open form. This activity disrupts the cavity holding the gold nanoparticle, releasing the encapsulated payload directly at the target site (Lo et al. 2010). Naturally, this principle can also apply to the TDN, opening the possibility for site‐directed, discreet nanoparticle delivery. Thus, while external hybridization remains a prevailing strategy for gene‐silencing applications, the exploitation of internal framework complementarity presents a transformative alternative for the directional transport and smart delivery of macromolecular therapeutic cargo.

### 2.3 Non‐Native Nucleotides and Linkages

**FIGURE 4:**
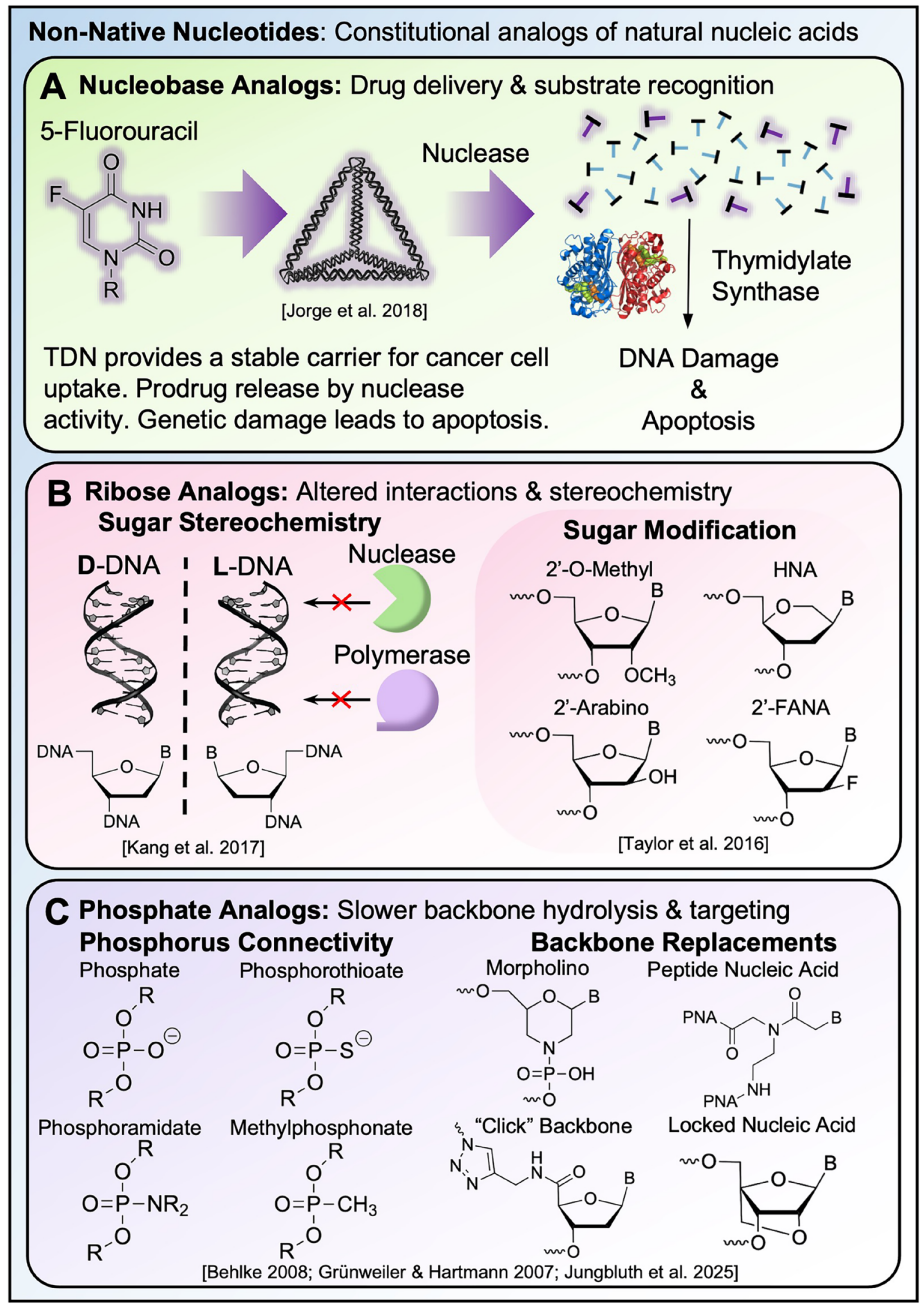
Preserving the canonical base‐pairing geometry while altering the chemical backbone provides profound pharmacokinetic controls. (A) Strategically modifying the nucleotide triad with therapeutic base analogs like 5‐fluorouracil for enhanced potency, (B) altered sugar rings to evade specific endonucleases, and (C) replacing phosphodiester bonds with more robust linkages maximizes the potency and systemic stability of the delivery vehicle without compromising base pairing.

To elevate the TDN from a purely structural carrier, researchers have successfully substituted the canonical nucleotides with therapeutic base analogs and stabilizing backbone modifications. Several nucleotide analogs have become synonymous with modern clinical applications of antisense and siRNA, but remain underrepresented in the TDN field (Burke et al. 2016; Sharma et al. 2014). Demonstrating this potential at the nucleobase, the direct sequence incorporation of the chemotherapeutic 5‐fluoro‐2′‐deoxyuridine transforms the TDN into a prodrug framework, relying on intracellular enzymatic hydrolysis to release the active payload (Jorge et al. 2018; Figure 4A).

Global stabilization can be achieved by altering the sugar conformation or components. Through the use of L‐DNA, an enantiomeric version of naturally occurring D‐DNA, the backbone can evade stereospecific endogenous nucleases (Kang et al. 2017). These mirrored deoxyribose sugars showed extended serum half‐lives from minutes to hours without changing the overall chemical composition (Figure 4B). Conversely, Taylor et al. (2016) showed that TDNs containing 2′‐fluoro, 2′‐fluoroarabino (FANA), and hexitol nucleic acids (HNA) could also assemble into resilient, thermally stable structures. They observed the HNA‐TDN remained fully intact for over 8 days while the unmodified TDN fully degraded in 48 h. Moreover, the stable FANA‐TDN perfectly mimicked the magnesium‐dependent folding of native DNA into B‐form duplexes (Taylor et al. 2016).

Completing this structural triad, the internucleotide phosphodiester linkage is the primary target for backbone stabilization (Figure 4C), as its susceptibility to enzymatic hydrolysis ultimately dictates the half‐life of the biopolymer (O'Reilly et al. 2026; Volk and Lokesh 2017). Substituting non‐bridging oxygens with sulfur to form a phosphorothioate (PS) backbone prioritizes nuclease resistance over native duplex stability (Conway et al. 2013; Behlke 2008). For example, Bai et al. demonstrated that a terminal 5′‐PS‐cap was insufficient to prevent degradation. However, total replacement of the phosphate backbone with PS granted their TDN unprecedented resistance to DNase I and Exonuclease III. This intrinsic modification drastically extended the operational lifespan of their TDN complex without interfering with detection of miR‐21 in living systems (Figure 3B; Bai et al. 2018).

Modifications such as locked nucleic acids (LNA) and peptide nucleic acids (PNA) are well established in linear antisense therapy. LNAs feature a 2′‐O, 4′‐C‐methylene bridge that rigidly locks the ribose sugar into a C3′‐*endo* conformation, dramatically enhancing target affinity and resistance to enzymatic hydrolysis (Grünweiler and Hartmann 2007; Frieden and Orum 2008). Similarly, PNAs utilize an uncharged pseudo‐peptide backbone, eliminating electrostatic repulsion and leading to exceptional hybrid duplex stability even in magnesium‐deficient environments (Jungbluth et al. 2025). While the structural incorporation of LNA into the TDN remains entirely uncharted, an early proof‐of‐concept exists for PNA integration (Zhang, Ma, et al. 2018). Ultimately, these advanced synthetic backbones represent a transformative and largely unexplored frontier for the TDN. However, the long‐term human safety and metabolic fate of novel xenobiotic nucleotide modifications remains largely uncharacterized. The chemical alterations that define the TDN as a highly customizable, responsive material also introduce a distinct milieu of structure‐related, hybridization‐independent toxicities. Decades of clinical evaluation with approved first‐generation PS oligonucleotide therapeutics reveal that their polyanionic nature drives nonspecific protein binding. Clinically, these effects manifest as significant dermal irritation or inflammatory lesions at the injection site (Levin 1999). The clinical translation of TDNs thus presents hidden risks when overengineered platforms eventually succumb to intracellular enzymatic cleavage.

## 3 Conjugation Strategies

### 3.1 Supramolecular Intercalation

The incubation of the TDN with discrete small molecules allows for non‐covalent payload integration, a process systematically categorized into three distinct topological binding mechanisms: intercalation, metallo‐coordination, and groove binding. Placing these interactions within a historical framework, numerous bioorganic approaches have sought to master the three‐dimensional assembly of multiple specific non‐covalent bonds in aqueous media (Dervan 2001).

**FIGURE 5:**
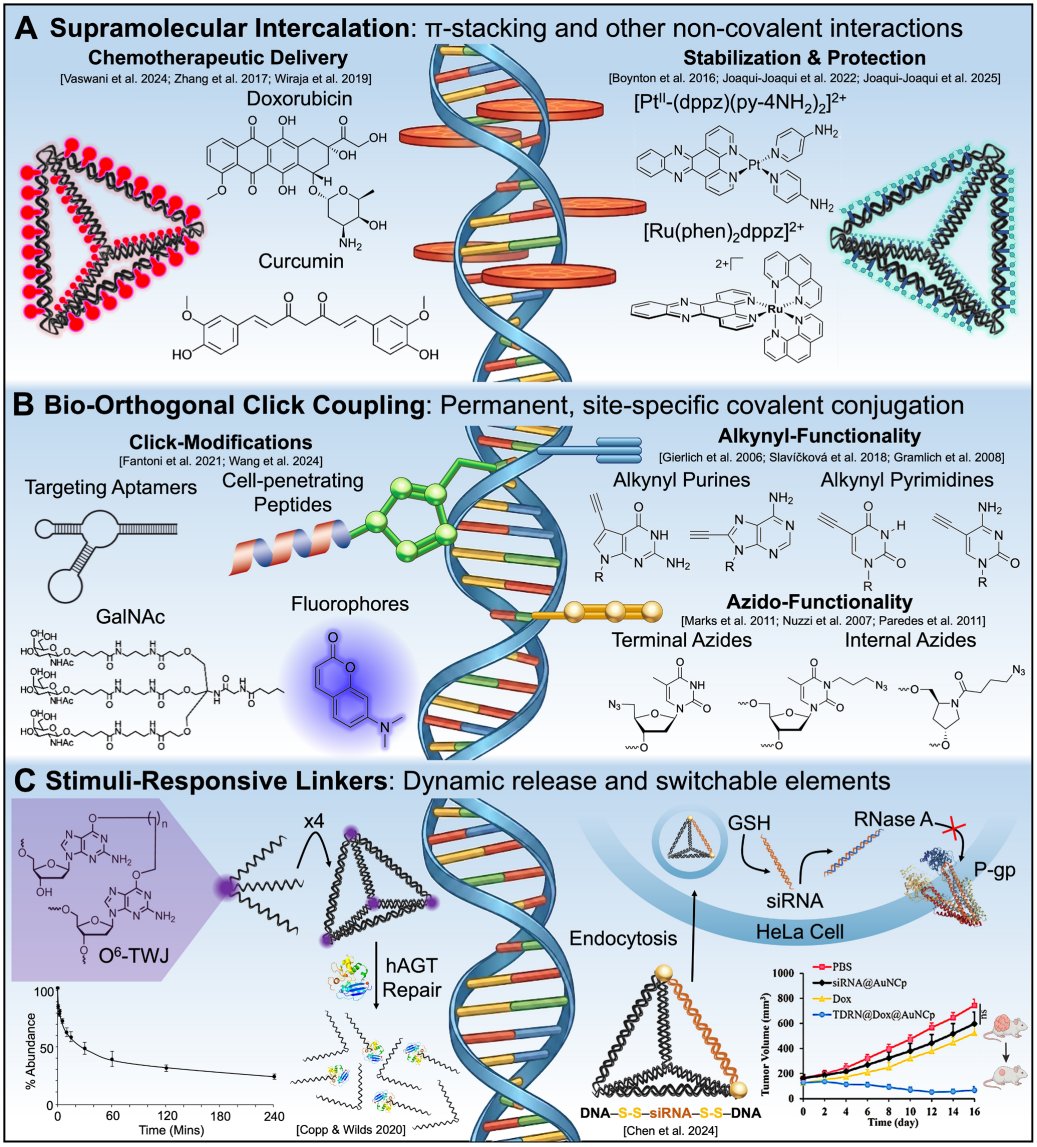
Approaches for therapeutic cargo loading and release from the TDN. (A) Intercalation of small molecules and metal complexes into the TDN scaffold provides a platform for chemotherapeutic drug loading and coordinated biochemical armor. (B) The CuAAC reaction can be used to couple various functional moieties. Conjugation occurs at specific sites using modified nucleotide units incorporated during oligonucleotide synthesis. (C) Covalent linkers with stimuli‐responsive elements can be unhooked by specific endogenous agents. The O6‐dG dimer is a substrate for the DNA repair protein hAGT (left). Disulfide‐linked siRNA releases from the scaffold through reduction by GSH in lysosomes (right).

The most prevalent strategy is intercalation, driven by the insertion of planar aromatic systems between the stacked nucleobases of the DNA duplex (Figure 5A). A principal example is the chemotherapeutic doxorubicin (DOX), a polycyclic aromatic compound routinely employed in the treatment of various aggressive cancers (Johnson‐Arbor and Dubey 2026; Xia and King 2025; Jones and Dass 2022). Given this drug's high systemic toxicity, the DOX‐TDN complex has emerged as a highly effective delivery vehicle demonstrating efficient loading and prolonged in vivo retention (Sinha et al. 2025). By fundamentally altering the pharmacokinetic profile of the free drug, the DOX‐TDN significantly enhances cellular uptake of the drug and reduces off‐target cytotoxicity (Vaswani et al. 2024; Zhang et al. 2017). Crucially, the morphology of the TDN directly governs this therapeutic efficacy. The physical diameter of the scaffold regulates the tissue penetration rate and loading capacity. For example, TDNs under 17 nm effectively penetrated the dermis up to 350 μm deep, achieving twofold greater drug accumulation and tumor inhibition relative to topically applied free‐DOX (Wiraja et al. 2019).

The processes of metallo‐intercalation and coordination chemistry leverage transition metal complexes to enhance both diagnostic delivery and structural fortification. The foundational biophysics of this interaction were established in photophysical and competitive binding studies with the polycyclic aromatic ligand dipyrido[3,2‐a:2′,3′‐c]phenazine (dppz) (Holmlin et al. 1998; Boynton et al. 2016). The resulting complex, [Ru(phen)_2_ dppz]^2+^, acts as a molecular “light switch” that is quenched in aqueous environments, but emits brightly when DNA‐bound (Holmlin et al. 1998). By creating the sterically hindered complex [Ru(Me_4_phen)_2_dppz]^2+^ it was possible to target mismatches, with 26‐fold stronger binding affinity than matched base pairs (Boynton et al. 2016). This evolved the coordination complex into a highly specific diagnostic tool for detecting cancer‐associated DNA mismatches. Recently, these principles have been repurposed to physically stabilize the TDN scaffold itself. While initial proof‐of‐concept designs relied on chiral ruthenium complexes (Joaqui‐Joaqui et al. 2022), subsequent iterations in 2025 employed achiral platinum and europium complexes to isolate the protective effects without enantiomeric interference (Joaqui‐Joaqui et al. 2025). This enhanced stability is driven by a unique dual‐binding mechanism: the dppz ligand intercalates into the major groove, while the 4‐aminopyridine ancillary ligands interact with the minor groove. This simultaneous occupancy enables a higher loading density, thermal stability, and nuclease resistance (Joaqui‐Joaqui et al. 2025).

Beyond intercalation, minor and major groove binding offers a complementary strategy driven by hydrogen bonding, Van der Waals forces, and the electrostatics of the phosphodiester backbone. This mechanism has even been harnessed by nature in the polyamide antibiotic distamycin A (Arcamone et al. 1964). Derivatives of this pyrrole chain have been used in the treatment of cancer by blocking transcription factors in the nucleus (Kurmis et al. 2017) and the mitochondria (Hidaka et al. 2017). Curcumin and its derivatives have been studied for their anti‐inflammatory (Fu et al. 2008), antimicrobial (Dai et al. 2022), and anticancer activity (Kunnumakkara et al. 2008). However, their clinical utility is severely hampered by poor aqueous solubility and rapid physiological degradation. By sequestering the payload within the grooves of the TDN, Chen et al. (2025) achieved a 20‐fold reduction in the required therapeutic dose. This stems from the TDN's ability to form a physical barrier, isolating curcumin from the external environment. These organic cargoes are notoriously susceptible to oxygen and light, and the restricted conformational space protects the drug from photoisomerization or oxidation. The groove binding also limits contact between these hydrophobic compounds, inhibiting possible aggregation and precipitation (Chen et al. 2025). Additionally, both major and minor groove‐binding modalities have been explored with potential applications in photodynamic therapy and targeted phosphodiester cleavage, respectively (Sánchez et al. 2019; Molphy et al. 2018). Ultimately, these three distinct non‐covalent modalities transform the native TDN from a passive framework into a highly versatile, multi‐payload carrier.

### 3.2 Bio‐Orthogonal Click Chemistry

One of the most rapidly expanding methods for the post‐synthetic functionalization of TDNs relies on bio‐orthogonal click chemistry (Figure 5B), through the copper‐catalyzed azide‐alkyne cycloaddition (CuAAC) or the strain promoted azide‐alkyne cycloaddition (SPAAC) reactions. This strategy allows for the precise, post‐synthetic incorporation of sensitive or sterically bulky modalities that would otherwise be difficult to integrate by the harsh conditions and shifting pH of automated DNA synthesis (Valsangkar et al. 2019; Seo et al. 2003; Qiu et al. 2013; Burley et al. 2006). The robust chemical stability of the triazole ring provides a permanent linkage for oligonucleotide conjugates (Cardona‐Galeano et al. 2025). By incorporating nucleotide phosphoramidites modified with reactive azide or alkyne functional groups, researchers can create modular docking ports for chemoselective bioconjugation. The spatial requirements of the payload dictate which modified nucleotides must be used. For sequence‐internal modifications, the canonical base is substituted with an analog such as 5‐ethynyluracil (Gierlich et al. 2006; Slavíčková et al. 2018; Jao and Salic 2008) or 5‐alkynylcytosine (Gramlich et al. 2008; Kuznetsova et al. 2023). To preserve the Watson–Crick binding face and minimize steric clashes, the backbone can be directly modified as well. These click handles can be chemical linkers or nucleotides, and can be positioned internally or terminally, allowing for even greater control of the bottom‐up engineering (Marks et al. 2011; Nuzzi et al. 2007; Isobe et al. 2008; Paredes and Das 2011). The modularity of the click reaction has made it pivotal in the rapid prototyping of covalently conjugated therapeutics and bioanalytical sensors (Fantoni et al. 2021).

In a seminal example of this chemistry, a TDN was constructed as a highly targeted drug delivery vehicle for colon cancer. The alkynyl termini of constituent single‐stranded DNA were clicked to DOX and folic acid molecules modified with azide groups. This bio‐orthogonal approach drastically enhanced membrane penetration and apoptosis rates compared to free‐DOX (Zhang et al. 2017). Building on this paradigm of modular targeting, a recent study pushed the boundaries of multifunctional delivery platforms. The authors utilized the CuAAC reaction to attach the p28 cell‐penetrating peptide directly to the 5′‐terminus of a TDN component strand before assembly. This peptide‐conjugated strand was then annealed alongside other functionalized strands containing an S6 targeting aptamer, a Cy5 imaging fluorophore, and a therapeutic siRNA, before finally being loaded with the intercalating chemotherapeutic daunorubicin. The resulting construct successfully increased non‐small cell lung cancer targeting and achieved a nearly 28% relative improvement in cytotoxicity compared to the free‐daunorubicin treatment. Furthermore, their data confirmed a significant decrease in off‐target effects in other cancers and virtually no uptake in normal cells (Wang, Chen, et al. 2024).

Beyond targeted therapeutics, the remarkable chemoselectivity of click reactions has also revolutionized bioanalytical diagnostics. Recent innovations have exploited target‐triggered cycloadditions to assemble catalytic DNAzyme sensors (Ye et al. 2025), while copper‐free SPAAC has been utilized to conjugate TDNs to metal–organic frameworks for the ultrasensitive luminescent detection of microRNA (Li et al. 2026). While these irreversible triazole linkages are ideal for constructing stable diagnostic interfaces and rigid delivery chassis, the targeted delivery of nanomedicine frequently demands dynamic, environmentally triggered payload release.

### 3.3 Stimuli‐Responsive Linkers

To address the limitations of permanent conjugation, researchers have increasingly integrated stimuli‐responsive linkers into the TDN architecture. The ultimate goal of these dynamic systems is to engineer nanodevices that actively respond to environmental cues. By replacing static covalent linkers with stimuli‐responsive moieties, researchers can program the TDN to undergo controlled disassembly or payload release only upon reaching a specific subcellular destination or interacting with a unique biomarker (Mossalam et al. 2010).

Exploiting the fundamental pH gradients present in the tumor microenvironment (pH 6.5) and the endosomal pathway (pH 5.5–4.5), pH‐responsive linkers offer a ubiquitous, endogenous trigger for payload release. The predictable nature of these conjugates underlines their capacity to autonomously trigger payload release without the need for exogenous activation. Simple responsive elements like hydrazones (Sonawane et al. 2017), acetals (Gillies et al. 2004), and imines (Tao et al. 2018) provide important acid‐labile linkages while more complex architectures like the i‐motif (Han et al. 2021) can be built into the scaffold. However, a critical weakness is the markedly narrow differential between physiological pH (~7.4) and the target site. Acid‐labile covalent linkages often suffer from premature hydrolytic cleavage during prolonged systemic circulation, resulting in off‐target or “leaky” release dynamics. Furthermore, structural pH triggers can introduce unwanted steric bulk or alter the thermodynamic stability of the TDN scaffold itself. To mitigate systemic vulnerabilities and enhance delivery, a recent TDN design has explored a lysosome‐activated i‐motif at the vertex. Gao et al. (2022) discovered that their pH‐responsive nanobox‐siRNA offered significantly faster endosomal release compared to siRNA delivered in liposomes. Both systems provided a similar concentration of siRNA to the cell over a 24‐h period; however, the nanobox showed significantly faster cellular uptake and localization from confocal fluorescent microscopy images. The deployment of these architectures requires a delicate balance between securing systemic stability and rapid, target‐specific intracellular release.

One of the most reliable physiological triggers is the cellular redox gradient. The cytosol of healthy eukaryotic cells maintains a high concentration of glutathione (GSH, 1 – 2 mM), nearly 1000‐fold higher than in the extracellular matrix (Forman et al. 2009; Lin et al. 2024). Furthermore, to combat the immense oxidative stress of rapid proliferation, cancer cells often sustain GSH concentrations upwards of 10 mM, allowing for the differentiation of these cells by redox‐responsive elements (Liu et al. 2023). This divergence accelerates reductive cleavage within the tumor microenvironment, offering a robust chemical trigger for targeted intracellular cargo release. Demonstrating this principle, Chen et al. introduced the tetrahedral DNA–RNA nanocage (TDRN), where the therapeutic cargo, an siRNA targeting the drug‐efflux pump P‐gp, is not attached as a pendant tail but is physically woven into the TDRN edge via disulfide bonds (Figure 5C). Unlike sequence overhang designs where exposed single‐stranded siRNA is rapidly degraded, embedding the RNA within the rigid edge confers exceptional stability, resisting RNase A degradation for over 90 min and remaining stable in serum for 12 h. Upon endocytosis and entry into the reducing environment of the cancer cell, the disulfide bonds are cleaved, triggering the disassembly of the edge duplex to release the functional siRNA, silencing P‐gp expression and thereby reversing evolved multidrug resistance (Chen et al. 2024).

While redox triggers rely on broad cellular gradients, enzymatic triggers allow TDNs to target‐specific cell phenotypes by exploiting endogenous proteins to actively dismantle the structure. Copp et al. designed a modified TDN that could undergo disassembly by the human repair protein O6‐alkylguanine DNA alkyltransferase (hAGT), which is frequently upregulated in chemotherapy‐resistant tumors (Figure 5C). To achieve this, standard phosphodiester bonds at the vertices were replaced with a noncanonical O6‐alkylene‐dG intrastrand cross‐link (IaCL). The hAGT protein recognizes this IaCL as a mutagenic alkyl lesion and attempts to repair it by irreversibly transferring the alkyl chain to a nucleophilic cysteine residue within the active site. This “repair” permanently severs the cross‐link holding the vertex together, causing the TDN to disassemble (Copp and Wilds 2020). This design has the potential to effectively turn a tumor's own resistance mechanism (DNA repair) into a self‐destruct trigger for the loaded nanocarrier.

While the described endogenous triggers rely on the cell's internal metabolic state, photo‐responsive linkers offer a distinct, extrinsic authority over the TDN behavior. This paradigm was established by Han et al. who incorporated azobenzene moieties directly into the DNA backbone to create a dynamic, “breathing” TDN. By exploiting the reversible stereo‐isomerization of azobenzene, they demonstrated that UV irradiation induces a *trans*‐to‐*cis* transition that contracts the structure by closing a hairpin, while visible light reverses this process to extend the scaffold (Han et al. 2011). This was later applied to cargo delivery in an engineered dual‐functional system combining click chemistry with a photocleavable linker (PCL). By integrating a nitrobenzyl‐based PCL alongside a 2′‐O‐propargyl attachment site, the UV‐triggered release of a fluorescein payload from the TDN was achieved. Notably, this system reached > 75% release efficiency within 4 min of exposure, validating the PCL as a trigger for liberating cargo on demand (Valsangkar et al. 2019). Recently, Peng et al. advanced this technology into the realm of intracellular diagnostics. Rather than facilitating simple cargo release, they utilized a PCL to physically block the toehold of a catalytic hairpin assembly probe attached to the TDN. Upon cellular uptake and localized UV activation (365 nm), the blocker is cleaved, allowing the system to sense miRNA‐10b with a detection limit of 28 pM. This photosensitive mechanism effectively eliminated nonspecific background noise, enabling high‐contrast imaging that successfully differentiated breast cancer cells (MCF‐7) from normal cells (MCF‐10A) based on their miRNA signatures (Peng et al. 2025).

## 4 Advanced Derivatives for Material Science

### 4.1 AFM Chemical Sensors

**FIGURE 6:**
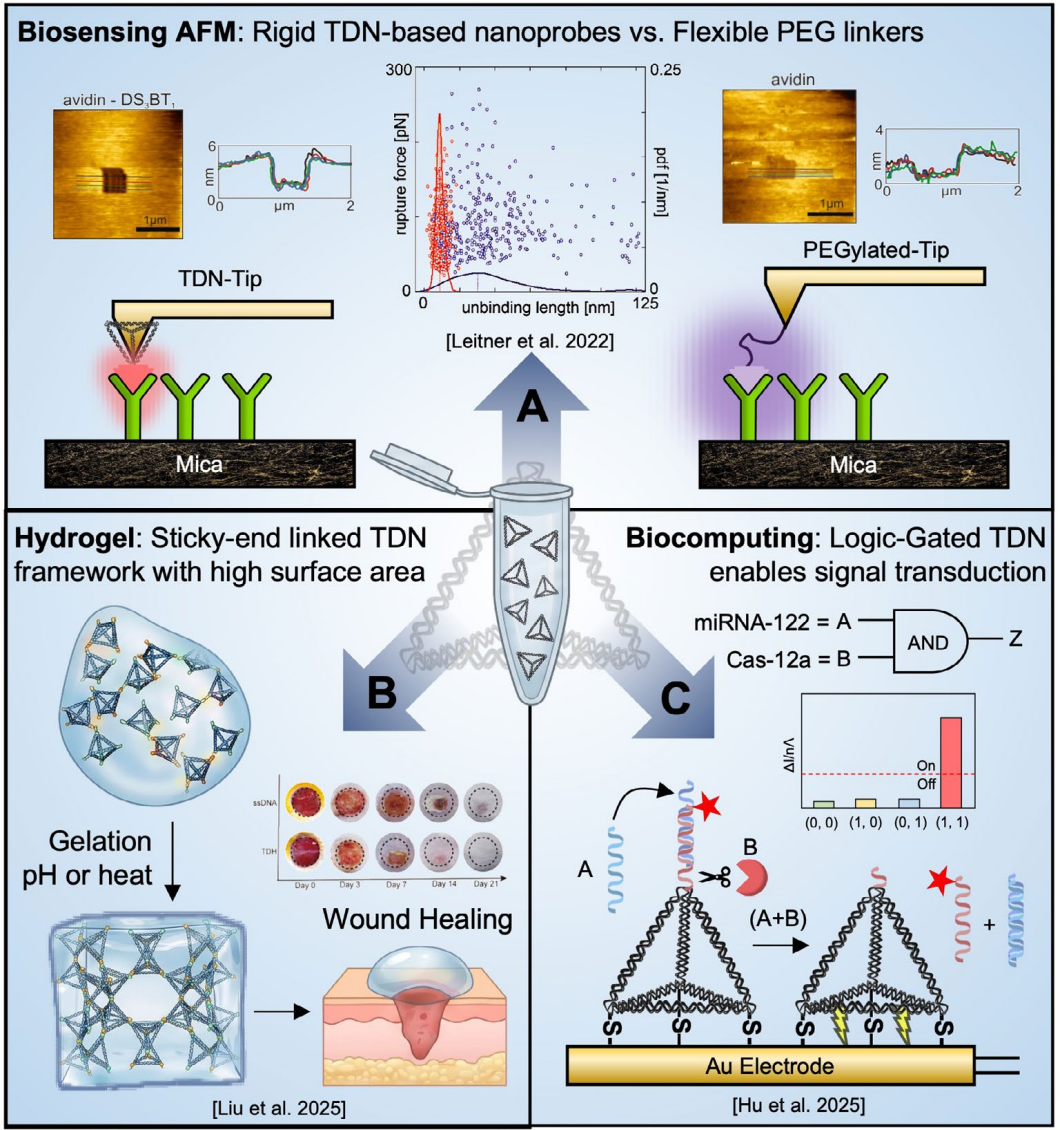
Versatility of TDNs in advanced biotechnological applications. (A) Comparison of AFM tip functionalization methods. The TDN provides a rigid attachment with consistent tether length. (B) TDN hydrogel frameworks assemble from solution phase. Application to burns in mice showed 33% faster wound healing. (C) DNA signal probe used for AND logic gate. Strand A competitively hybridizes with the dark‐blue strand, while CRISPR/Cas12a recognizes and cleaves the ssDNA strand. Loss of Fc‐label from ssDNA reduces current and amplifies the signal.

While the majority of TDN research focuses on molecular processes, a novel physical application has emerged in the field of Atomic Force Microscopy (AFM), utilizing the TDN not as a carrier, but as a rigid interfacial linker. Traditionally, to transform an AFM tip from a simple topographic probe into a chemical biosensor required complex silanization of the probe or the use of flexible polyethylene glycol (PEG) tethers to attach sensing molecules (Headrick and Berrie 2004; Dong and Sahin 2011; Sedlak et al. 2020). However, the inherent disorder of polymer linkers introduced a “broad distribution of rupture lengths” during AFM, obscuring the precise biophysical mechanics of the ligand–receptor interaction (Leitner et al. 2022). To address this limitation, they introduced a strategy utilizing the TDN as a rigid, tetra‐functional DNA linker that precisely orients the gold‐coated tip and the sensing molecule (Figure 6A). Three engineered vertices with disulfide anchors bind the structure to the tip, while the fourth vertex presents sensing aptamers or biotin to the substrate surface. The flexible PEG linker produced a diffuse rupture profile of 26.4 ± 6.2 nm, while in direct comparison, the rigid TDN provided a defined rupture event at 8.7 ± 0.8 nm. This represents a significant improvement in accuracy and precision for sub‐nanometer, single‐molecule recognition events. Furthermore, their method demonstrated exceptional shelf‐stability, effectively allowing for the batch functionalization of multiple tips at the same time (Leitner et al. 2022). This streamlined and automatable protocol proves critical for biosensing AFM, transitioning the technique from a specialized physical chemistry tool into a robust, unambiguous platform suitable for advanced spatial diagnostics.

### 4.2 Tetrahedral Hydrogel Frameworks

Advancements in our understanding of how the TDNs assemble into nanomaterials have led researchers to innovate beyond rigid geometry and construct macroscopic materials (Ko et al. 2020). In a striking departure from standard payload‐delivery models, Liu et al. recently repurposed the TDN as a fundamental structural monomer. Two unique TDNs were prepared with complementary sticky ends, allowing for the assembly of a 3D DNA hydrogel (Figure 6B). Inspired by the continuous tetrahedral lattice of a diamond, the macroscopic DNA network exhibited exceptional self‐healing and mechanical strength, boasting a storage modulus 8.6‐fold higher than conventional DNA hydrogels. Crucially, the hydrogel was deployed in murine studies for severe burn wound healing without the inclusion of a pharmaceutical payload. The dense, porous material inherently scavenged reactive oxygen species from the wound, providing rapid hemostasis while attenuating the local inflammatory cascade. The pure hydrogel successfully reprogrammed macrophages into an anti‐inflammatory phenotype, ultimately accelerating tissue regeneration in vivo (Liu et al. 2025). This work establishes a novel frontier for TDNs, proving that their highly ordered structures can yield bulk biomaterials with profound intrinsic therapeutic properties.

### 4.3 Biochemical Computing

Beyond its fundamental biological role in genetic programming, DNA has emerged as a unique medium for high‐density data storage and molecular computing. In another radical departure from traditional applications, researchers have increasingly leveraged the extreme mechanical rigidity of TDNs to construct nanoscale biocomputers and diagnostic interfaces (Figure 6C). The predictable thermodynamics of Watson–Crick base pairing can be engineered into biochemical circuits capable of executing complex logic operations, neural network simulations, and dynamic algorithmic processes. When parallel computing strands are densely tethered to a surface, they frequently suffer from severe steric hindrance, intermolecular entanglement, and sluggish reaction kinetics, severely degrading computational accuracy (Hu et al. 2025). To overcome these spatial limitations and bridge the gap between solution‐phase computing and solid‐state biological hardware, researchers are leveraging the defined spatial organization of TDNs. Exploiting this structural advantage, Yang et al. recently constructed an electrochemical biosensor featuring a TDN‐based “AND” Boolean logic gate for the parallel detection of dual hepatocellular carcinoma (HCC) biomarkers. The system was partially driven by a toehold‐mediated strand displacement, such that the presence of miRNA‐122 unwound the double‐stranded signal probe at the vertex of an anchored TDN. The simultaneous presence of the protein alpha‐fetoprotein (AFP) in the surrounding environment activated a CRISPR/Cas12a enzyme. If both biomarkers were present to initiate their respective cascades, the “AND” logic gate would be activated, altering the electrochemical signal from the electrode. Because the TDN scaffold perfectly regulated the distance between probes and eliminated steric hindrance, the CRISPR/Cas12a complex could easily access the target. This structural precision resulted in exceptional diagnostic sensitivity, achieving limits of detection as low as 4.05 fg/mL for AFP and 34.84 aM for miRNA‐122, successfully distinguishing between the blood serum of healthy individuals and HCC patients (Yang et al. 2025).

## 5 Conclusions and Outlook

The advent of TDNs and related framework‐nucleic acids represents a pivotal shift from classical top‐down lithography techniques for molecular manufacturing. Reminiscent of the arrival of macroscale 3D printing, bottom‐up molecular self‐assembly allows matter to predictably organize into defined lattices. By capturing the inherent programmability of Watson–Crick base pairing, researchers have effectively transformed nucleic acids from the native biological operating system into engineered biomaterials. Functionalization of the TDN can be accomplished by an extensive array of covalent and non‐covalent approaches tailored to support the desired application (summarized in Table 1). Just as modern software runs on compiled code in silicon, future nanotechnology will leverage programmable polymers to dictate how matter organizes. This unparalleled structural precision grants molecular architects absolute stoichiometric control and sub‐nanometer spatial resolution. However, moving beyond elegant, in vitro proof‐of‐concept designs, firmly establishing TDNs as the cornerstone of next‐generation nanomedicine involves navigating a multitiered translational pipeline. This developmental trajectory spans three interdependent domains: AI‐driven drug discovery, rigorous chemistry, manufacturing, and controls (CMC), and in vivo clinical translation.

**TABLE 1:** Summary of conjugation strategies for DNA tetrahedra.

Conjugation type	Conjugation strategy	Advantages	Disadvantages	References
Preassembly covalent	Terminal‐end labeling via Phosphoramidite chemistry	Atomic precision, high yield, integration during strand synthesis.	Limited to small modifications, synthetic length constraints, chemical waste.	Roy and Caruthers (2013), Alvarado‐Urbina et al. (1981), Lacroix et al. (2019), Didenko (2001), Zhong et al. (2018), Theisen et al. (1992), Gaur (1991), Kupihár et al. (2003), Lu and Zhang (2018), Winkler (2015), Bergamini et al. (2014), Lietard et al. (2019)
	Nucleotide modifications Base, sugar	Superior nuclease stability, enhanced target binding affinity.	High synthesis cost, potential for structural distortion if overused.	Copp and Wilds (2020), Jorge et al. (2018), Zhang, Ma, et al. (2018), Burke et al. (2016), Sharma et al. (2014), Taylor et al. (2016), O'Reilly et al. (2026), Volk and Lokesh (2017), Behlke (2008), Bai et al. (2018)
	Backbone replacements Phosphate	Charge neutralization, modulates biodistribution, preserves Watson–Crick base pairing.	Technically challenging synthesis, potential for in vivo hydrolysis.	Kang et al. (2017), Grünweiler and Hartmann (2007), Frieden and Orum (2008), Jungbluth et al. (2025)
Stable covalent	Click chemistry (CuAAC/SPAAC)	Bio‐orthogonal, highly efficient, preserves sensitive therapeutic payloads.	Requires pre‐functionalization (azide/alkyne), potential steric hindrance for large payloads, high risk of degradation by copper.	Xia et al. (2016), Wang et al. (2020), Valsangkar et al. (2019), Seo et al. (2003), Qiu et al. (2013), Burley et al. (2006), Cardona‐Galeano et al. (2025), Gierlich et al. (2006), Slavíčková et al. (2018), Jao and Salic (2008), Gramlich et al. (2008), Kuznetsova et al. (2023), Marks et al. (2011), Nuzzi et al. (2007), Isobe et al. (2008), Paredes and Das (2011), Fantoni et al. (2021), Wang, Chen, et al. (2024), Ye et al. (2025), Li et al. (2026)
	Nucleophile/electrophile NHS, maleimide, hydrazone	Established protocols, mild coupling conditions, many commercially available functionalities.	Rapid hydrolysis of NHS esters in basic conditions, maleimide linkages are susceptible to thiol exchange, high risk of off‐target coupling.	Sonawane et al. (2017)
Dynamic covalent	Disulfide Tunable redox potential	Logic‐gated release via redox switches (GSH), highly specific to the tumor microenvironment.	Risk of premature cleavage in blood, complex synthesis protocols.	Chen et al. (2024), Pontarelli et al. (2022), Leitner et al. (2022)
	pH‐responsive linkers Acid or base labile	Acid‐triggered release targeting endosomes or tumor extracellular matrix.	pH variability among patients, stability issues during prolonged circulation.	Han et al. (2021), Sonawane et al. (2017), Gillies et al. (2004), Tao et al. (2018), Gao et al. (2022)
	Enzyme‐cleavable linkers Site‐specific stimuli	Enzyme‐triggered release targeting specific tissues or cellular organelles.	Complex, multistep synthesis required to integrate cleavable moieties, linker must be sterically accessible to protein.	Copp and Wilds (2020), Jorge et al. (2018), Lietard et al. (2019), Li et al. (2025), Molphy et al. (2018), Yang et al. (2025)
Non‐covalent	Intercalation Pi‐stacking and groove binding	No chemical modification required; simple loading process; reversible binding.	High risk of premature leakage, limited to specific planar/aromatic molecules or metal complexes.	Bai et al. (2018), Grünweiler and Hartmann (2007), Frieden and Orum (2008), Jungbluth et al. (2025), Levin (1999), Dervan (2001), Johnson‐Arbor and Dubey (2026), Xia and King (2025), Jones and Dass (2022), Sinha et al. (2025), Vaswani et al. (2024), Zhang et al. (2017), Wiraja et al. (2019), Holmlin et al. (1998), Boynton et al. (2016), Joaqui‐Joaqui et al. (2022), Arcamone et al. (1964), Kurmis et al. (2017), Hidaka et al. (2017), Fu et al. (2008)
	Sticky‐end hybridization Watson–Crick and Hoogsteen	Plug‐and‐play modular docking site, rapid prototyping without resynthesis of scaffold.	Potential for nonspecific hybridization, highly susceptible to pH, temperature and serum nucleases, relies on relatively weak hydrogen bonding.	Lee et al. (2012), Schlapak et al. (2012), Bu et al. (2013), Desai and Shankar (2003), Conway et al. (2013), Longmire et al. (2008), Nguyen et al. (2019), Faheem et al. (2022), Huang et al. (2024), Goodman et al. (2008), Li et al. (2019)

### 5.1 AI‐Driven Design

**FIGURE 7:**
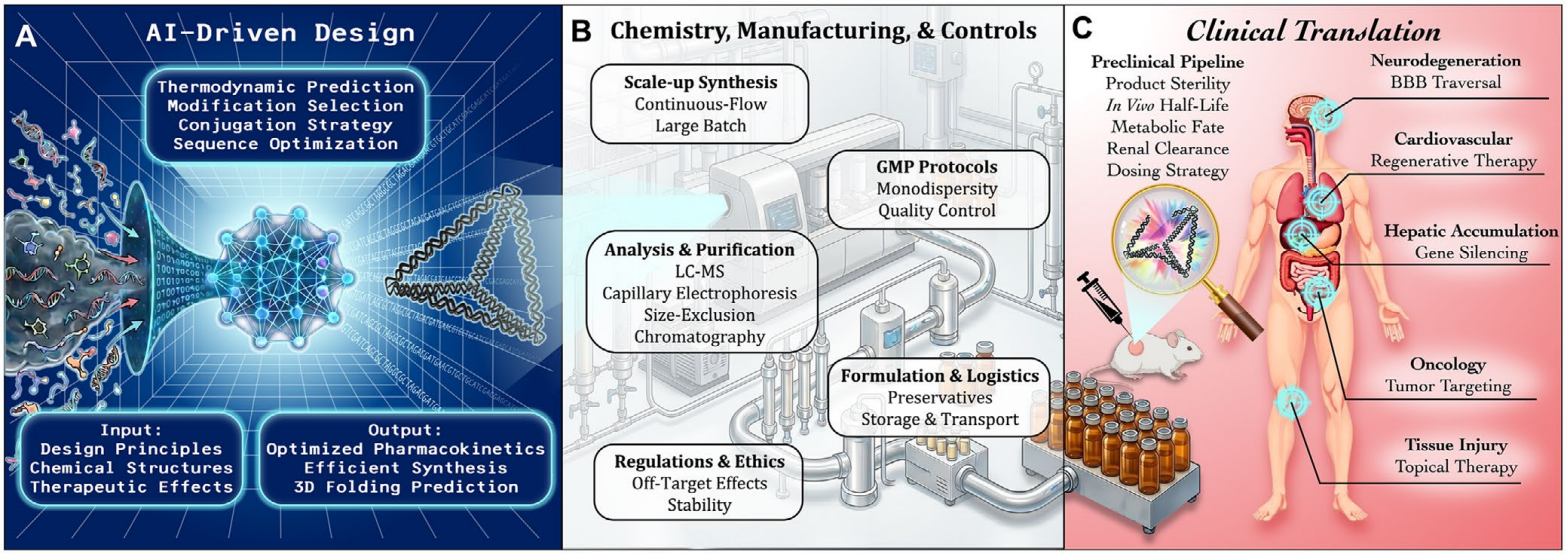
Emerging frontiers for the TDN in medicine. (A) Modern advancements in artificial intelligence provide unparalleled screening capabilities to optimize all aspects of a TDN before it reaches the benchtop. (B) Practical scale‐up protocols must be validated rigorously while cold‐chain logistics are established. (C) Evaluating the performance of DNA nanotechnology long‐term in vivo and key hurdles to clinical translation.

The initial bottleneck in TDN optimization lies in the near‐infinite sequence design space. Sequence permutations expand exponentially according to the power law *X* ^*n*^, where “*n*” represents the polymer length and “*X*” denotes the monomeric units available. Even small frameworks present astronomical combinatorial options, before beginning to consider noncanonical modifications. Many studies generate sequences based on algorithms, such as *caDNAno*, that screen for nonspecific complementarity and structure homogeneity (Douglas et al. 2009; Amoako et al. 2013; Veneziano et al. 2016), but frequently neglect critical chemical and biological variables. Human intuition alone is insufficient to navigate this vast combinatorial matrix. Accordingly, the integration of advanced artificial intelligence systems with next‐generation drug design will become standard practice (Figure 7A). The ability to rapidly generate and screen expansive libraries of theoretical constructs, execute molecular dynamics simulations, and optimize conjugates is at the frontier of computational biology. This digital front‐end serves as the predictive engine, evaluating the nanostructure before physical synthesis and validation ever begins.

### 5.2 Chemistry, Manufacturing, and Controls

Once optimized on the benchtop, the practical hurdles of physical scale‐up synthesis and rigorous quality control begin. Transitioning TDNs from milligram‐scale batches to kilogram‐scale commercial production demands a shift to automated continuous flow or large‐batch reactors (Figure 7B). Following process scale‐up, achieving absolute monodispersity under strict Good Manufacturing Practices (GMP) remains an ongoing challenge (Andrews et al. 2021). Truncated oligonucleotide species, structural abnormalities, and endotoxin contamination must all be minimized before the first batches are cleared for human trials (Corey et al. 2022). Resolving these manufacturing pitfalls requires the integration of robust, high‐throughput analytical validation and purification pipelines. Biophysical techniques such as dynamic light scattering (DLS), AFM, and high‐resolution LC–MS must be deployed to profile batch‐to‐batch fidelity. Industrial scale capillary electrophoresis and preparative size‐exclusion chromatography are required to clear trace synthesis impurities and nonspecific annealing aggregates (Corey et al. 2022). Furthermore, the long‐term commercial viability of TDN therapies hinges on strategic formulation and cold‐chain logistics. To incentivize biopharmaceutical adoption and clinical translation, the economics must align with cost‐effective manufacturing (Corey et al. 2022). As a novel macromolecular platform, ensuring prolonged structural integrity will require the development of specialized preservatives, validated with rigorous, long‐term storage and transport simulations. Collectively, this extensive CMC infrastructure represents a mandatory threshold that must be crossed before the drug reaches the clinic.

### 5.3 Clinical Translation

Despite the undeniable elegance of these framework nucleic acids, early‐phase human clinical trials present stark physiological realities. While TDN‐based therapeutics possess the clear potential to disrupt the nanomedicinal landscape (Figure 7C), significant translational bottlenecks regarding pharmacokinetics and toxicological safety remain unresolved. Preclinical pipelines must comprehensively map the metabolic fate, serum half‐life, distribution, and renal clearance pathways of these complex assemblies. Beyond clearance by the immune system, the biological firewalls encountered upon intravenous administration force an unavoidable operational trade‐off between absolute structural persistence and efficient intracellular bioavailability (Chandran et al. 2025). Furthermore, the structural complexity of modified TDNs introduces an unprecedented metabolic profiling challenge. Unlike the predictable derivatives of small‐molecule drugs, a single decomposed TDN yields a heterogeneous population of thousands of intermediate‐sized fragments and modified monomers. Each case presents distinct toxicological profiles: High‐molecular weight fragments can exceed the threshold for efficient renal filtration, leading to rapid accumulation and subsequent nephrotoxicity. Oligonucleotide fragments, between 10 and 25 nt, can act as unintentional antisense agents. If these sequences are not thoroughly cross‐referenced against the human genome during the design phase, they risk triggering nonspecific gene silencing. Liberated mononucleotides are inherently xenobiotic when modified for specific therapeutic effect. If the compounds are not recognized and expelled, they can infiltrate the host cell's nucleotide salvage pathway. These artificial monomers risk incorporation into nuclear or mitochondrial DNA, potentially driving unintended mutagenesis, oncogenesis, or apoptosis, reversing any potential benefits of the original therapy.

### 5.4 Realization and Convergence

Successfully navigating these barriers will unlock the true multimodal potential of nucleic acid scaffolds. Precisely engineering the edges and vertices of the tetrahedron with combinations of cell‐penetrating peptides, aptamers, or ligands enables true modular therapies. This spatial versatility facilitates targeted vectors for neuro‐regeneration via blood–brain barrier (BBB) traversal (Shen et al. 2026), cardiovascular regenerative therapy (Zhang et al. 2019), localized oncological gene therapy (Cheng et al. 2022), or sustained topical delivery for tissue repair (Huang et al. 2026). Ultimately, surviving the translational “valley of death” requires radical scientific convergence. Pushing TDNs past intricate research chemicals into validated, human‐grade therapeutics necessitates extensive computational foresight, scalability in manufacturing, and complete characterization within human physiology.

## Author Contributions

**Tyler J. Rutherford:** writing – original draft, conceptualization. **Christopher J. Wilds:** conceptualization, writing – review and editing, supervision, funding acquisition.

## Funding

This work was supported by the Natural Sciences and Engineering Research Council of Canada (NSERC) Discovery Grant Program (RGPIN‐2023‐04821 to C.J.W.), NSERC Collaborative Research and Training Experience Program (CREATE, CREATE 528279‐2019) in Programmed Molecules for Therapeutics, Sensing and Diagnostics (PROMOTE), NSERC and Fonds de recherche du Québec—Nature et technologies (FRQNT) graduate scholarship programs.

## Conflicts of Interest

The authors declare no conflicts of interest.

## Data Availability

Data sharing is not applicable to this article as no datasets were generated or analyzed during this study.
